# Supplementary material for: Wake slow waves in focal human epilepsy impact network activity and cognition
Source: Nat Commun. 2023 Nov 30;14:7397. doi: 10.1038/s41467-023-42971-3 (PMC10689494; doi:10.1038/s41467-023-42971-3)
Supplement: Supplementary file 1 — Supplementary Information [file 41467_2023_42971_MOESM1_ESM.pdf]

Supplementary Tables and Figure

Wake slow waves in focal human epilepsy impact network activity and cognition

Age at recording	Handedness	Gender	Hemisphere	Duration recording (min)	Expected Seizure focus
38	L	M	L	21	mesial temporal
34	R	M	L	10	temporal/MRI negative
37	R	F	R	9	temporal
33	R	M	B	23	temporal
41	R	F	L	26	frontal
51	R	M	L	12	temporal
47	R	M	L	33	temporo-occipital
35	R	M	L	13	temporal

Age at recording	Handedness	Gender	Hemisphere	Hipp	Amyg	neoT	Duration recording (min)	Expected Seizure focus
38	A	M	L	4	2	10	46	Hippocampus
44	R	F	L	2	3	4	35	Anterior Hippocampus
42	R	M	R	1	2	7	51	Mesial Temporal Lobe
59	R	M	R	4	0	4	79	Temporal Neocortex
51	R	F	L	6	0	6	51	Mesial Temporal Lobe
47	R	F	R	3	0	15	218	Mesial Temporal Lobe
27	R	M	R	4	0	5	37	Posterior Parahippocampal Gryus
41	R	F	R	2	2	8	49	Anterior Frontal Cortex
22	R	M	L	2	1	9	98	Occipito-temporal Cortex
43	R	M	L	2	3	11	160	Could not be determined
26	R	M	R	2	2	8	149	Middle Frontal Gyrus
38	R	F	R	3	2	5	82	Frontal Cortex / Insula
37	R	F	R	6	3	9	81	Frontal Cortex
26	R	F	L	5	3	9	153	Posterior Cingulate
39	R	F	L	1	3	5	250	Amygdala
22	R	F	R	4	0	3	20	Fusiform gyrus
44	R	M	R	7	2	8	25	Frontotemporal

Supplementary Table 1 | Patients demographics

Top Demographics of patients included for microelectrodes recordings. *Bottom* Demographics of patients included for macroelectrodes recordings. Hipp=hippocampal contacts. Amyg=amygdala contacts. NeoT=neocortex temporal contacts. L=left. R=right. A=ambidexter. B=bilateral. M=male. F=female.

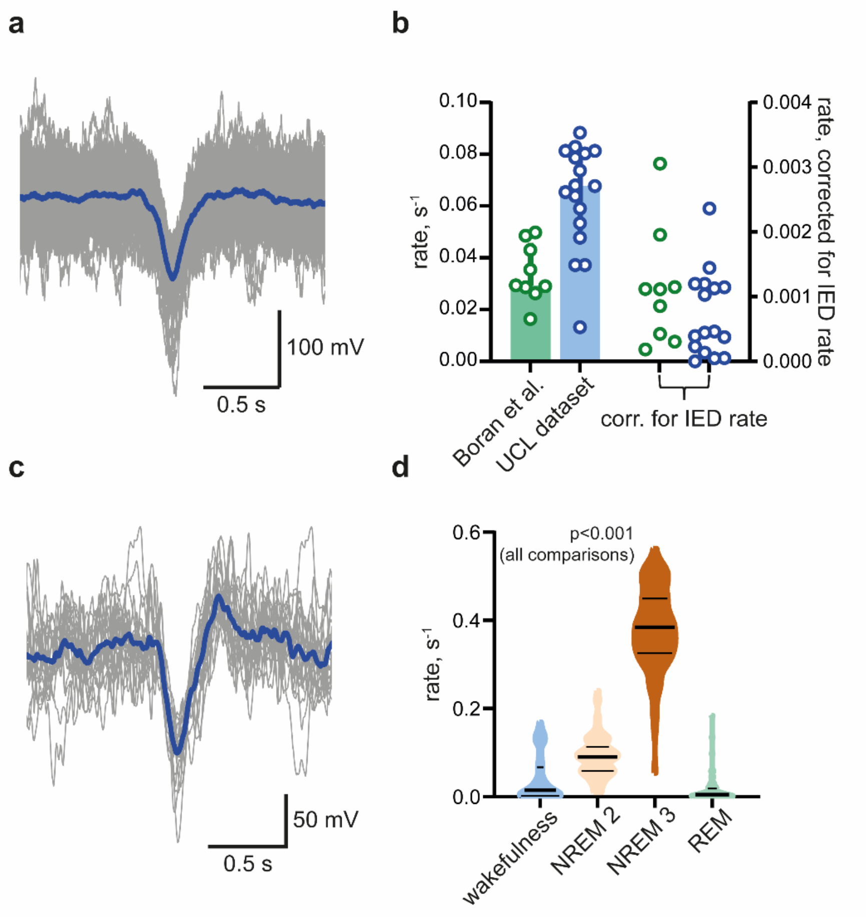

Supplementary Fig. 1 | Wake slow waves can be identified in independent datasets and are rarer during wakefulness than sleep.

(a) Example of wake slow waves identified in one patient with epilepsy from another centre (from Boran et al., 2020). **(b)** The events identified in the Boran dataset were rarer than in our cohort (first two columns, unpaired two-sided permutation t-test, p=0.0004, n=9 and n=17 patients in the Boran and UCL datasets respectively). However, after correcting for differences in the incidence rate of interical epileptiform discharges (IEDs) (see Fig. 3f), the rate was similar (last two columns, unpaired two-sided permutation t-test, p=0.22, n=9 and n=16 patients in the Boran and UCL datasets respectively). **(c)** Example of wake slow waves identified in one patient with epilepsy from a third centre (MNI Open iEEG Atlas). **(d)** When SW are detected with the same amplitude threshold across the sleep-wake cycle, those detected during wakefulness are significantly less frequent than during NREM 2 and NREM 3 (mixed-effect analysis, F(1.83, 212.8)=680.3, p<0.0001, mean ± SD during wakefulness: 0.04 s^{-1} $electrode^{-1}$ ± 0.05, NREM stage N2: 0.09 ± 0.04, NREM stage N3: 0.38 ± 0.09, REM: 0.02 ± 0.03, Tukey's post-hoc analysis: all comparisons at p<0.0001 except wakefulness *vs* REM at p=0.0002, n=106 patients during wakefulness, n=91 during NREM 2, n=91 during NREM 3 and n=65 during REM). Violin plots show the frequency distribution of data; central bold line is median; thin lines above the median are quartiles.

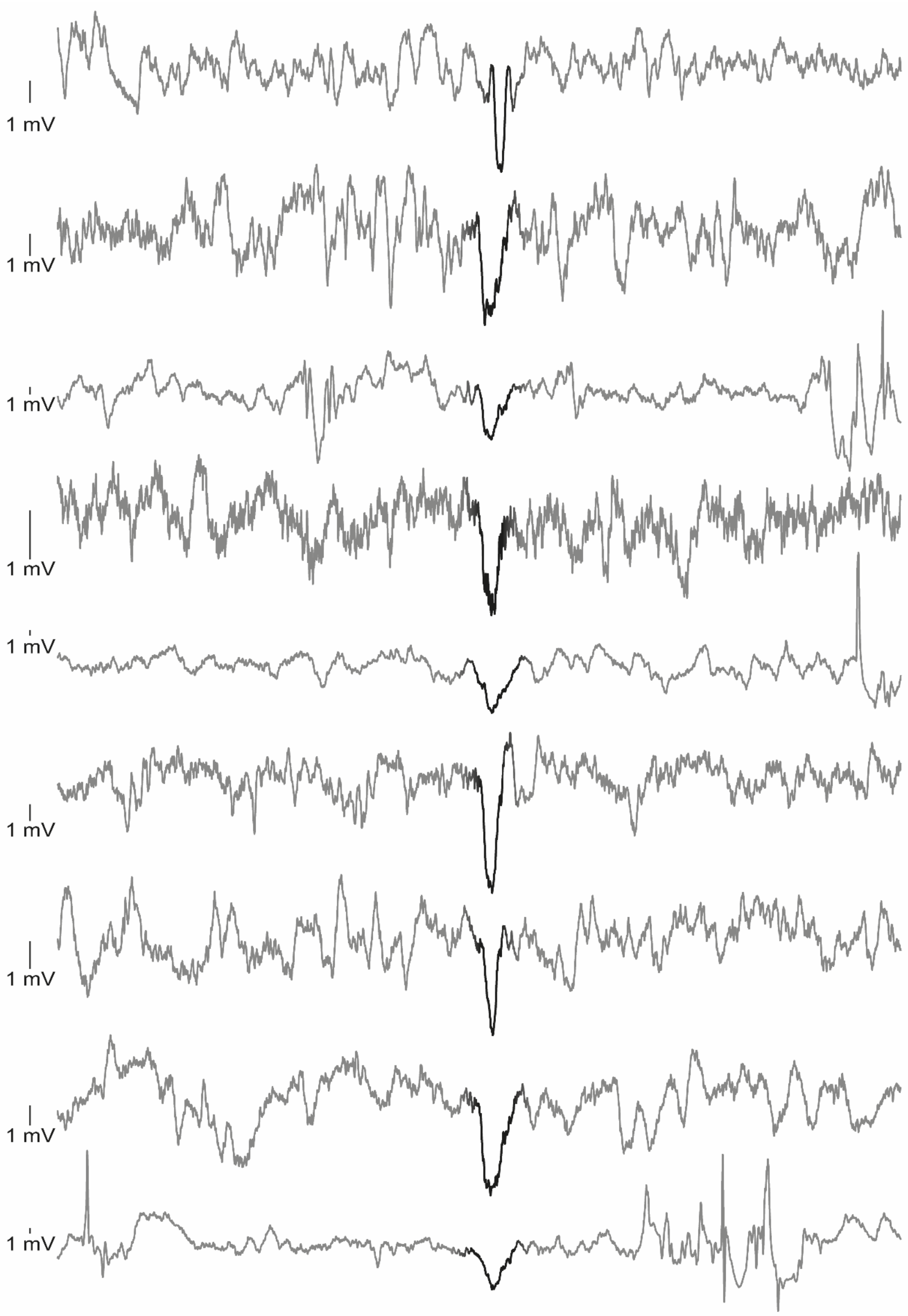

Supplementary Fig. 2 | Examples of local wake slow waves

Examples of local slow waves (SW) identified during wakefulness (raw local-field potential signal). Each window lasts 10 seconds and is centred on the onset of a detected SW.

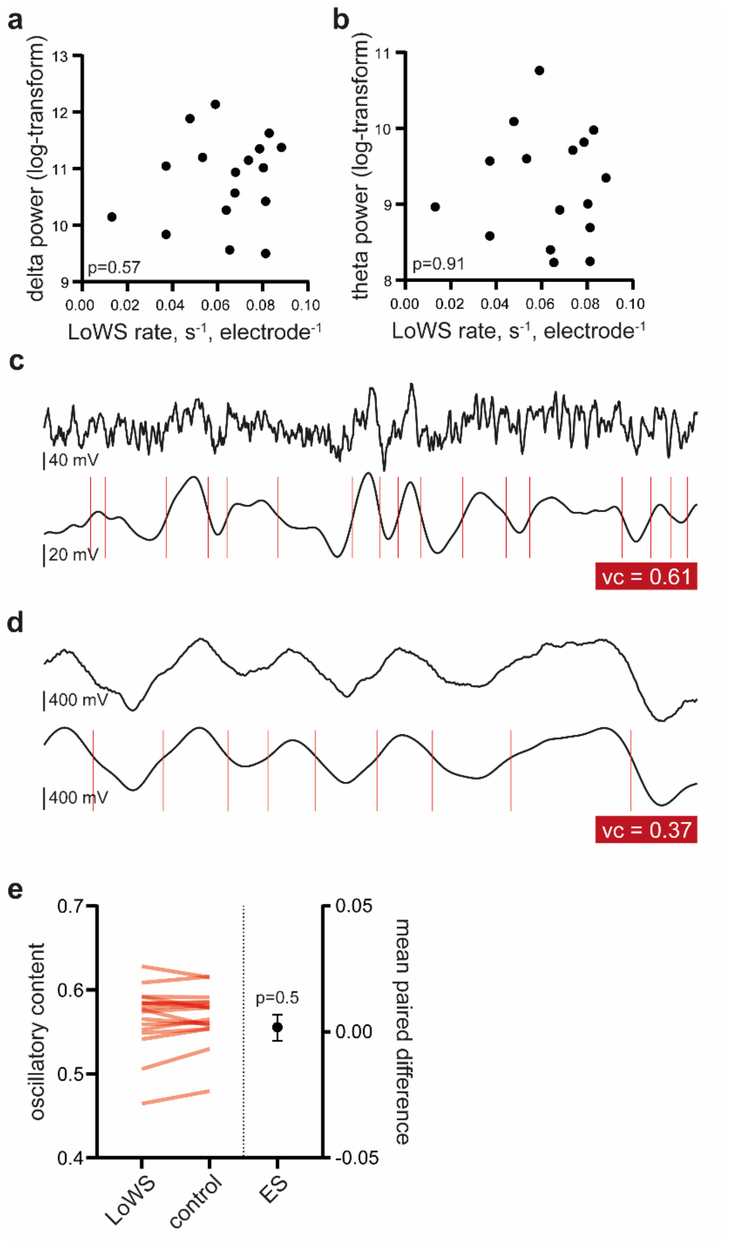

Supplementary Fig. 3 | Epileptic focus and slow waves

(a-b) There is no correlation between the rate of hippocampal wake slow waves and delta (a) or theta (b) power (Pearson correlation, r^2=0.02, two-sided p=0.57 and r^2=0.0009, two-sided p=0.91 respectively, n=17 patients in both correlations). **(c-d)** Estimates of the oscillatory content in a period without (c) and with (d) oscillatory activity. The raw signal (top of each panel) is filtered within the frequency band of interest (bottom of each panel, here 0.5-4 Hz). Each zero-crossing is then identified (red vertical bars). Finally, the coefficient of variation (CV) of the delay between each successive zero-crossing is used as an estimate of the oscillatory content (CV is high in the absence of, and low in the presence of, an oscillation). **(e)** The oscillatory content is similar around LoWS and during background (control) activity (two-sided paired permutation t-test, p=0.501, n=17 patients, ES expressed as mean and 95% CI). ES = effect size; LoWS = local wake slow waves.

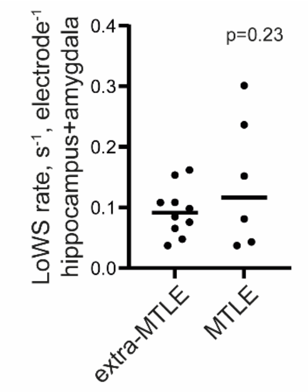

Supplementary Fig. 4 | LoWS do not co-localize with the epileptic focus
LoWS occur with similar incidence rates on mesial temporal lobe (MTL) electrodes in patients with extra-mesial temporal lobe epilepsy (extra-MTL) and MTL (unpaired permutation t-test, two-sided p=0.226, n=10 patients with extra-mesial and n=6 patients with mesial temporal lobe epilepsy). Patient #10 is not included because of unclear location of the epileptic focus. Bars: median.

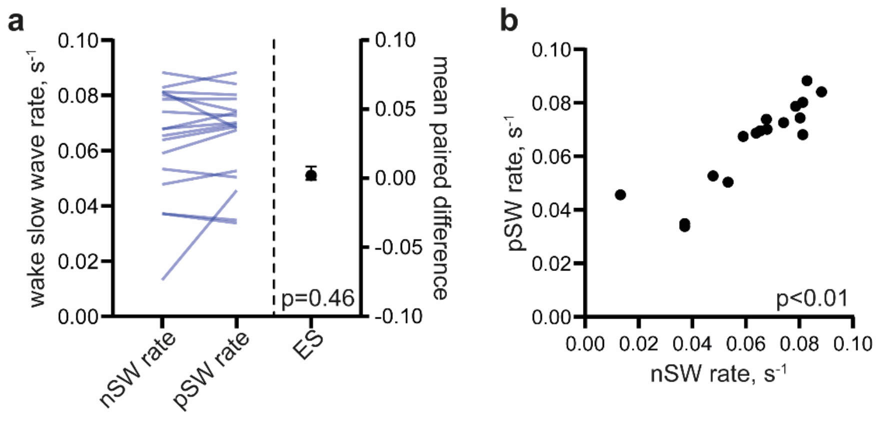

Supplementary Fig. 5 | nSW and pSW rate

(a) Waves with negative (nSW) and positive (pSW) polarity occur at the same incidence rate (two-sided paired permutation t-test, p=0.464, n=17 patients, ES expressed as mean and 95% CI). **(b)** nSW are strongly correlated with pSW across patients (Pearson correlation, r^2=0.79, two-sided p<0.0001, n=17 patients). ES=effect size.

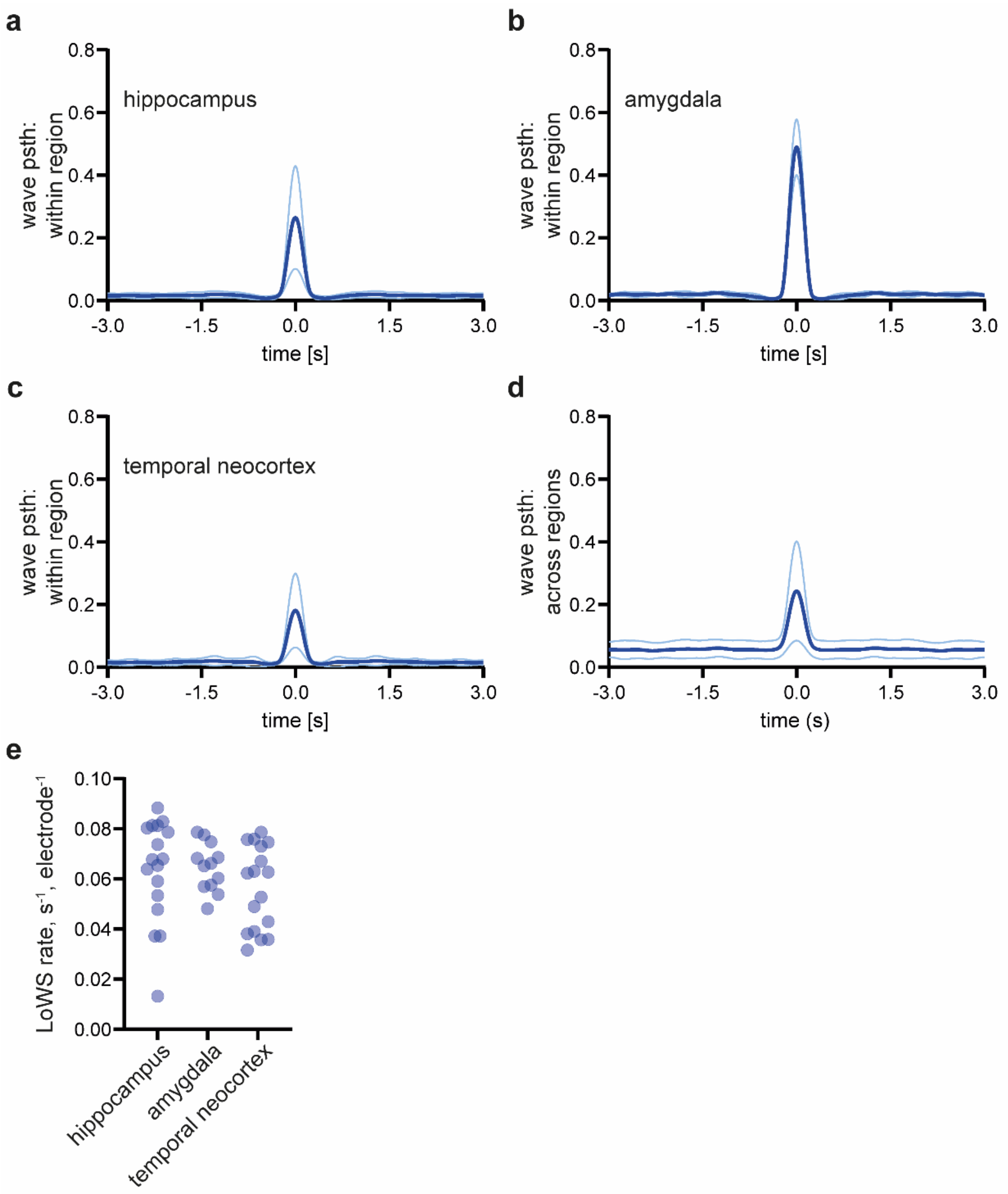

Supplementary Fig. 6 | Focality and micro-focality of LoWS

(a-c) When a LoWS is identified on a specific contact in the hippocampus (a), 27% of other hippocampal contacts will record a LoWS. This co-occurrence rate reaches 49% in the amygdala (b) and 18% in the temporal neocortex (c). Traces are mean ± SD (n=15 patients for hippocampus, n=11 patients for amygdala and n=17 for temporal neocortex) **(d)** When a LoWS is identified in one brain region, it will be also identified in one of the two other regions in 24% of cases (n=17 patients). **(e)** The rate of LoWS per second, per electrode, is similar between the hippocampus, the amygdala and the temporal neocortex.

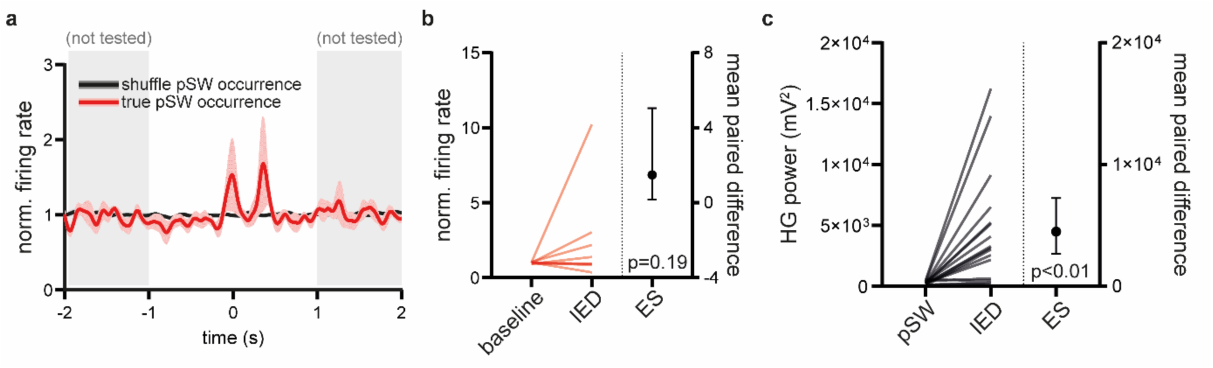

Supplementary Fig. 7 | High-gamma and neuronal firing around pSW and IEDs

(a) Mean firing rate ± SEM around pSW peak (peak at 0 s). There is no significant difference from baseline (estimated by firing rate around randomized pSW occurrence, black trace). **(b)** Mean firing rate around IEDs ([-0.05 to 0.05 s]) shows only a non-significant increase trend (two-sided paired permutation t-test, p=0.19, n=8 patients). ES=effect size. **(c)** High-gamma (HG, 45-130 Hz) power is significantly higher during IEDs than pSW (when taking either the window of analysis of IEDs: [-0.05 to 0.05 s] around IEDs and pSW or the window of analysis of LoWS: [-0.15 to 0.165 s] around IEDs and pSW, two-sided paired permutation t-test, p=0.0008 for both time-windows, n=16 patients). Here, HG power is measured from -0.15 to 0.165 s around IEDs and pSW peak.

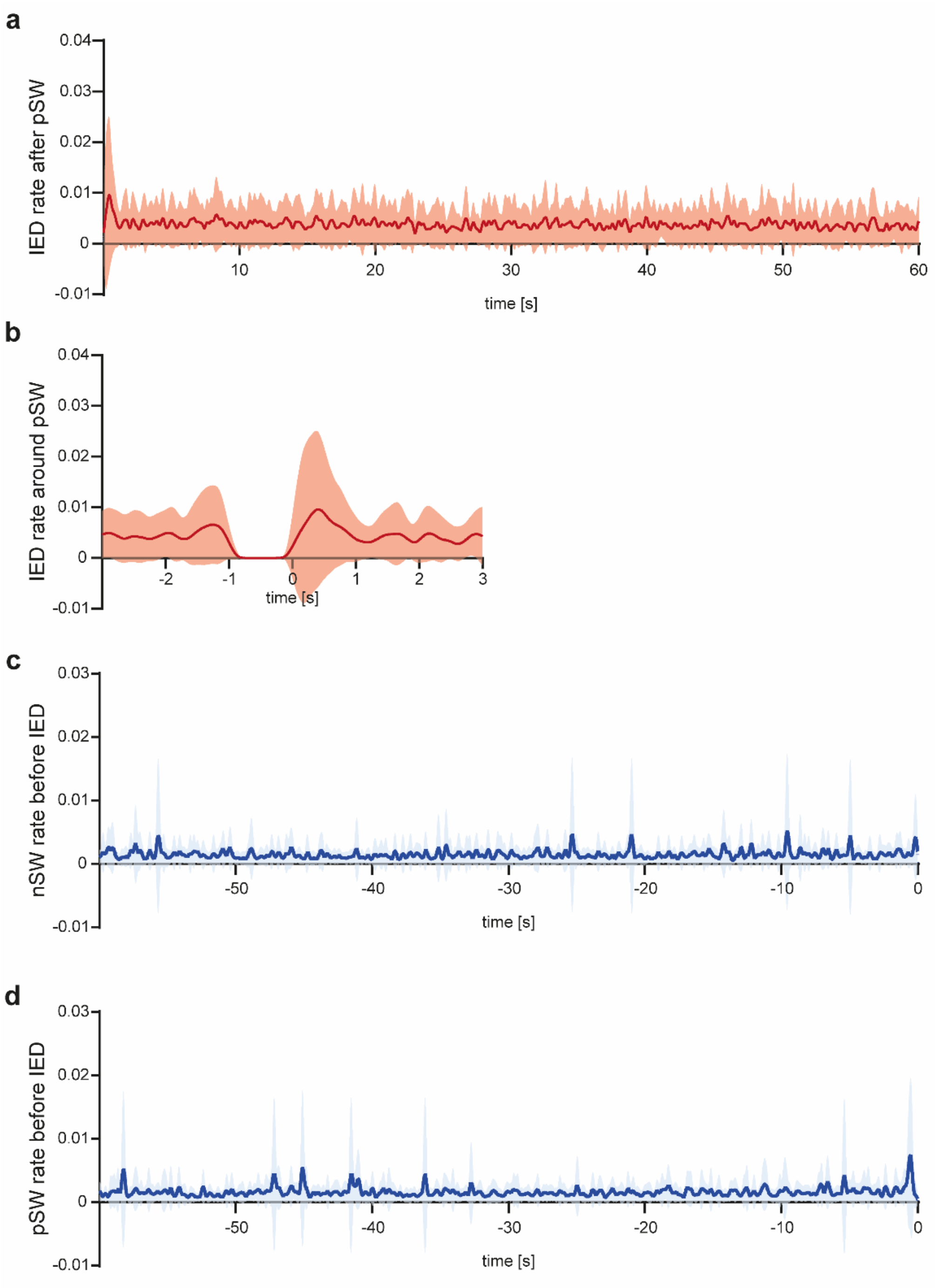

Supplementary Fig. 8 | pSW do not activate IEDs

(a-b) The presence of a SW with positive polarity (pSW) is not associated with a long-term (a) or short-term (b) change in the incidence rate of IEDs (per s, per electrode and per pSW). The x-axis is the time relative to the occurrence of a wake SW. Traces are mean ± SD, n=16 patients. **(c-d)** Similarly, the incidence rate of nSW (c) and pSW (d) do not change before IEDs. The x-axis is the time relative to the occurrence of an IED. Traces are mean ± SD, n=16 patients.

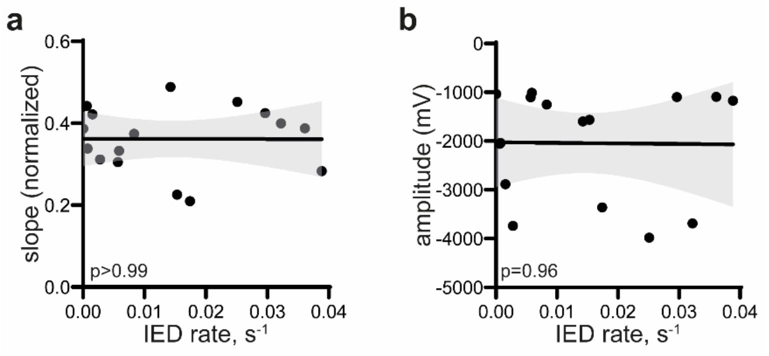

Supplementary Fig. 9 | No correlation between IEDs rate and LoWS slope, nor amplitude

(a) There is no correlation between LoWS slope and IED rate (Pearson correlation, two-sided *p*>0.99, n=16 patients). **(b)** There is no correlation between LoWS amplitude and IED rate (Pearson correlation, two-sided *p*=0.96, n=16 patients). Shaded area is the 95% confidence bands.

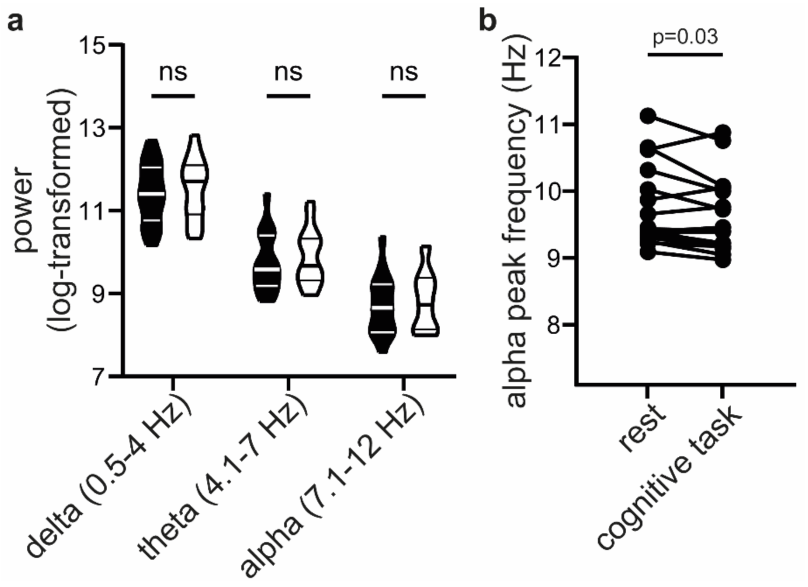

Supplementary Fig. 10 | Rest and cognitive task periods present the same spectral signatures

(a) Mean power in the delta (0.5-4 Hz), theta (4.1-7 Hz) and alpha (7.1-12 Hz) frequency bands during the task (white) and rest periods outside of the task (black) did not differ (2 way ANOVA, effect of condition [during vs outside task]: $F(1, 16)=3.827$, $p=0.0681$; interaction condition * frequency: $F(2, 32) = 0.1334$, $p=0.8756$, n=17 patients, violin plots with median and interquartiles), suggesting that patients' vigilance was not modulated by task demands. **(b)** Peak alpha frequency is slightly higher during rest (9.7 Hz) than during the cognitive task (9.6 Hz) (two-sided paired t-test, $p=0.0262$).

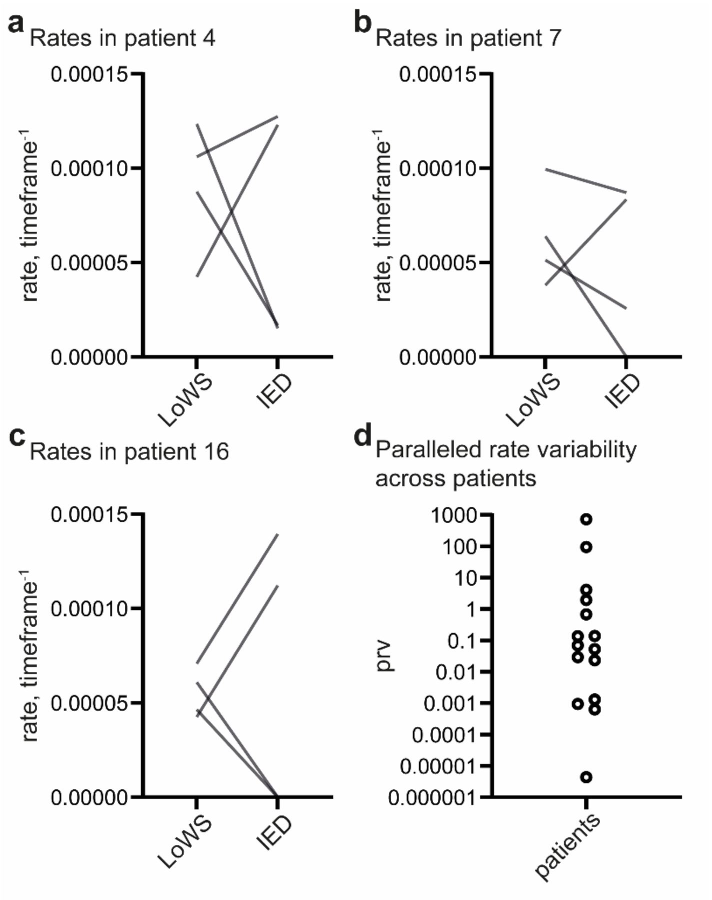

Supplementary Fig. 11 | Rate of LoWS and IEDs across electrodes

Among other verifications, to confirm that LoWS were not mere mislabelled IEDs, we tested whether the rates of LoWS and IEDs follow the same trend across electrodes. A parallel trend would be expected according to the null hypothesis (LoWS are mislabelled IEDs). Here we show 3 typical examples (**a-c**) of LoWS- (left in each panel) and IEDs-rate (right in each panel) in 3 different patients. In each panel, each line corresponds to one electrode. As seen, patients 4 and 16 have two electrodes that show a higher rate of IEDs than LoWS, and two that show a lower rate of IEDs than LoWS. Patient 7 has 3 electrodes that show a lower rate of IEDs than LoWS, and one that shows a higher rate of IEDs than LoWS. **(d)** Across patients, the parallel rate variability index ("prv", see Online Methods) was significantly different from 0 (one sample Wilcoxon test, two-sided $p<0.0001$, n=16 patients), reflecting that the rate of LoWS does not follow the rate of IEDs across electrodes.

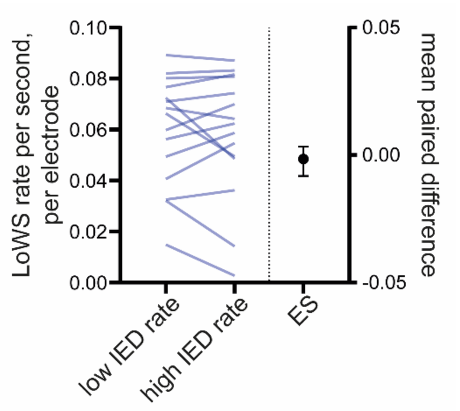

Supplementary Fig. 12 | LoWS rate at maximal IEDs rate

Following the analysis in Supplementary Fig. 11, another way to verify that LoWS are not mislabelled IEDs was to check whether electrodes recording the highest IEDs-rate are also those recording the highest LoWS-rate. As shown here, electrodes with the highest IEDs-rate have not a significantly higher rate of LoWS than all other electrodes (two-sided paired permutation t-test, p=0.595, n=15 patients, ES is mean and 95% CI). ES=effect size.

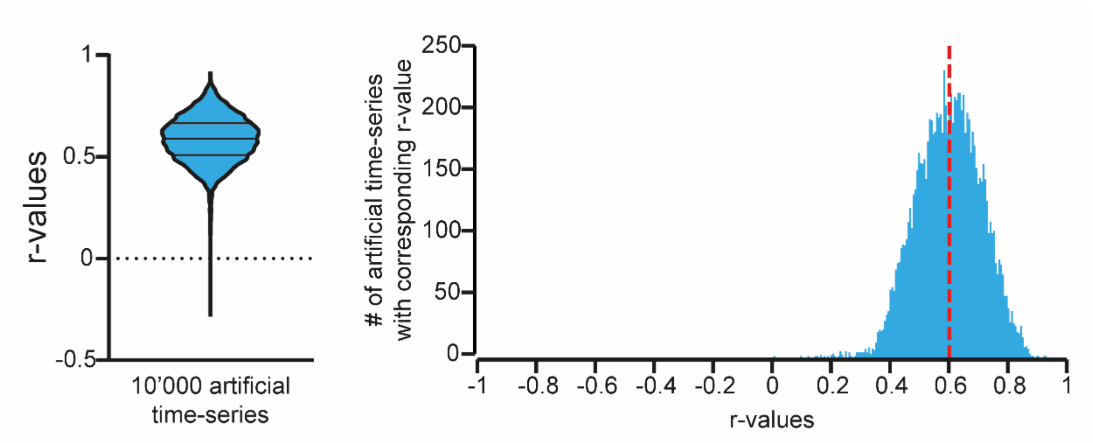

Supplementary Fig. 13 | Confirmatory analysis: mislabelled IEDs would be positively correlated with IEDs across patients

To demonstrate that mislabelled IEDs would be positively correlated with the incidence rate of IEDs across patients, we constructed 17 artificial time-series (i.e., as many as our cohort) which length was at least as long as our shortest EEG recording and at most as long as our longest EEG recording, and inserted a random number of markers (markers A), then changed the label in a random proportion (markers A'), and calculated the rate of these two subsets of markers and the correlation between them and across the 17 time-series. We repeated the operation a large number of times (10'000 times) to obtain a distribution of the *r*-coefficient. This distribution of *r*-values had a median value significantly above 0 (Wilcoxon one sample test, median, IQ: 0.59, 0.51-0.67, $p<0.0001$), confirming that the rate of one marker (A) and a subpopulation of markers made of mislabelled original markers (A'), are positively correlated. Violin plot: median and quartiles are shown. Red dashed line: median.

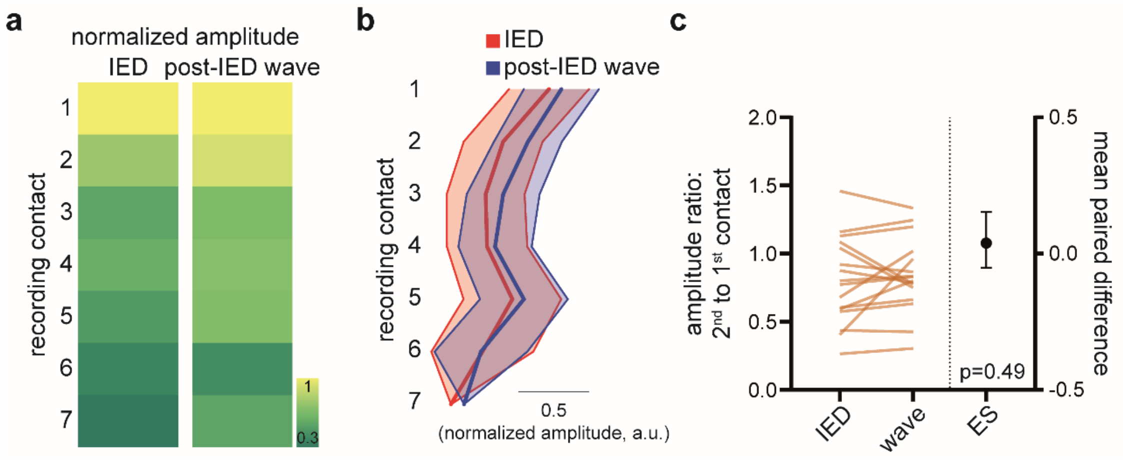

Supplementary Fig. 14 | Volume conduction similarly affects IEDs and post-IED waves
To address the concern that slow components of the EEG are more widely volume conducted than fast components, and therefore that the identified slow waves might reflect volume conducted post-IED waves in which the sharp component has not diffused, we measured **(a)** the normalized amplitude of the IED (at IED detection, i.e., maximal amplitude) and the post-IED wave (absolute maximal amplitude over the following 0.5 s) across recording contacts on each depth electrode and observed a comparable rate of decrease from the recording contact on which the IED was detected (top of each column in this example) to the most distal recording contact (bottom of each column). Color scale: amplitude, normalized to the maximal amplitude per patient. **(b)** Normalized, mean amplitude ± SD of IEDs and post-IED waves across depth electrode contacts, showing no significant difference. **(c)** Statistical comparison of the ratio of amplitude on the first to second contact for IEDs and post-IED waves ('wave'). No significant difference is observed (two-sided paired permutation t-test, *p*=0.49, n=16 patients, ES is mean and 95% CI). ES=effect size.
