# Supplementary material for: Wake slow waves in focal human epilepsy impact network activity and cognition
Source: Nat Commun. 2023 Nov 30;14:7397. doi: 10.1038/s41467-023-42971-3 (PMC10689494; doi:10.1038/s41467-023-42971-3)
Supplement: Supplementary file 3 — Reporting Summary [file 41467_2023_42971_MOESM3_ESM.pdf]

nature portfolio

Corresponding author(s): Prof. Matthew Walker

Last updated by author(s): Oct 23, 2023

Reporting Summary

Nature Portfolio wishes to improve the reproducibility of the work that we publish. This form provides structure for consistency and transparency in reporting. For further information on Nature Portfolio policies, see our Editorial Policies and the Editorial Policy Checklist.

Statistics

For all statistical analyses, confirm that the following items are present in the figure legend, table legend, main text, or Methods section.

n/a	Confirmed	
☐	☒	The exact sample size (*n*) for each experimental group/condition, given as a discrete number and unit of measurement
☐	☒	A statement on whether measurements were taken from distinct samples or whether the same sample was measured repeatedly
☐	☒	The statistical test(s) used AND whether they are one- or two-sided<br>*Only common tests should be described solely by name; describe more complex techniques in the Methods section.*
☐	☒	A description of all covariates tested
☐	☒	A description of any assumptions or corrections, such as tests of normality and adjustment for multiple comparisons
☐	☒	A full description of the statistical parameters including central tendency (e.g. means) or other basic estimates (e.g. regression coefficient) AND variation (e.g. standard deviation) or associated estimates of uncertainty (e.g. confidence intervals)
☐	☒	For null hypothesis testing, the test statistic (e.g. *F*, *t*, *r*) with confidence intervals, effect sizes, degrees of freedom and *P* value noted<br>*Give P values as exact values whenever suitable.*
☒	☐	For Bayesian analysis, information on the choice of priors and Markov chain Monte Carlo settings
☒	☐	For hierarchical and complex designs, identification of the appropriate level for tests and full reporting of outcomes
☐	☒	Estimates of effect sizes (e.g. Cohen's *d*, Pearson's *r*), indicating how they were calculated

Our web collection on statistics for biologists contains articles on many of the points above.

Software and code

Policy information about availability of computer code

Data collection	Acquisition: Micromed and Blackrock acquisition system. Software for analyses: Matlab (mainly, see Data analysis below).
Data analysis	Codes for slow wave and interictal epileptiform discharges detection are available on https://github.com/bushlab-ucl. Frequency analyses were performed using Fieldtrip codes (https://www.fieldtriptoolbox.org/). Single and multi-unit activity were identified using wave_clus (version 3.0.3, https://github.com/csn-le/wave_clus). We also used Matlab (v. 2022a), Graphpad Prism (v. 9 and updates, also for illustration), Adobe Illustrator (v. 26 and updates, for illustration only), SPSS (v. 29) and the web application EstimationStats (https://www.estimationstats.com/#/).

For manuscripts utilizing custom algorithms or software that are central to the research but not yet described in published literature, software must be made available to editors and reviewers. We strongly encourage code deposition in a community repository (e.g. GitHub). See the Nature Portfolio guidelines for submitting code & software for further information.

Data

Policy information about availability of data

All manuscripts must include a data availability statement. This statement should provide the following information, where applicable:

- Accession codes, unique identifiers, or web links for publicly available datasets
- A description of any restrictions on data availability
- For clinical datasets or third party data, please ensure that the statement adheres to our policy

This work is based on clinical data that was subject to ethics committee approval and patient consent. We will share clinical data on request, provided that request

fulfils the ethics approval that we have for data collection and analysis. Source data for the main Results are provided with the paper.
The MNI dataset is accessible on https://mni-open-ieegatlas.research.mcgill.ca/. The dataset is powered by LORIS which is under license GPLv3.
The Boran dataset is accessible on https://doi.gin.g-node.org/10.12751/g-node.d76994/. The Boran dataset is under license CC BY-SA 4.0 DEED.

Human research participants

Policy information about studies involving human research participants and Sex and Gender in Research.

Reporting on sex and gender	We collected sex data which is reported but could not be controlled for.
Population characteristics	Patients (n=25) with medically refractory epilepsy (11 female, 23 right-handed, mean age of 38.5 yrs, 17 with macroelectrode recordings and 8 with microelectrode recordings) undergoing intracranial EEG monitoring for clinical purposes at the National Hospital for Neurology and Neurosurgery, London
Recruitment	Patients were recruited if the research protocol did not impact negatively on their clinical care, and if intracranial electrodes were targeted at mesial temporal lobe structures.<br>Patients were recruited sequentially from one center (UCLH). They all had investigation of temporal lobe structures, so there could be some bias in that the temporal lobe might have been involved in the epileptic network. However, only a third had seizures actually originating in the mesial temporal lobe, but it would be unethical to place electrodes outside presumably epileptic networks. We have acknowledged this potential bias for the temporal lobe in the Methods (p. 5).
Ethics oversight	Prior approval was granted by the NHS Research Ethics Committee (15/LO/1783), and informed written consent was obtained from each patient

Note that full information on the approval of the study protocol must also be provided in the manuscript.

Field-specific reporting

Please select the one below that is the best fit for your research. If you are not sure, read the appropriate sections before making your selection.

☒ Life sciences ☐ Behavioural & social sciences ☐ Ecological, evolutionary & environmental sciences

For a reference copy of the document with all sections, see nature.com/documents/nr-reporting-summary-flat.pdf

Life sciences study design

All studies must disclose on these points even when the disclosure is negative.

Sample size	We are reporting a previously unreported phenomenon of unknown frequency and so initial sample size calculations were not possible. Given the rarity of human intracranial recordings, sample size was determined by the availability of the data.
Data exclusions	Data with artefactual activity that would have impeded a reliable analyses have been discarded. For this reason, one patient with microelectrode recording was excluded, making a total of 8 patients included in the analyses.
Replication	We used 2 independent datasets of patients with epilepsy, from 2 different centers, and who also benefitted from intracranial recording to verify the presence of slow wave during wakefulness. In both datasets, we could identify slow waves.
Randomization	No randomization was applied, since we performed mostly paired analyses. We recruited all patients who were suitable and consented to be part of the trial and we were not comparing patients. Each patient acted as his/her own control and the sample is not large enough to investigate the impact of covariates, such as age and sex. However, these were not relevant to the main aims of the study, i.e., determine whether wake slow waves were present and had a functional impact.
Blinding	Detection of slow waves was performed automatically with a post-processing verification to remove artefactual periods. For this reason, blinding was not performed. Subsequent analyses were also automatically performed.

Reporting for specific materials, systems and methods

We require information from authors about some types of materials, experimental systems and methods used in many studies. Here, indicate whether each material, system or method listed is relevant to your study. If you are not sure if a list item applies to your research, read the appropriate section before selecting a response.

Materials & experimental systems

n/a	Involved in the study
☒	☐ Antibodies
☒	☐ Eukaryotic cell lines
☒	☐ Palaeontology and archaeology
☒	☐ Animals and other organisms
☐	☒ Clinical data
☒	☐ Dual use research of concern

Methods

n/a	Involved in the study
☒	☐ ChIP-seq
☒	☐ Flow cytometry
☒	☐ MRI-based neuroimaging

Clinical data

Policy information about clinical studies

All manuscripts should comply with the ICMJEguidelines for publication of clinical research and a completedCONSORT checklist must be included with all submissions.

Clinical trial registration	Prior approval was granted by the NHS Research Ethics Committee (15/LO/1783)
Study protocol	There is no full trial protocol available (the study is not a clinical trial).
Data collection	Data were collected in the Epilepsy Monitoring Unit of Queen's Square Institute of Neurology, University College London, UK. Time of recording depended on clinical activity.
Outcomes	Given the design and questions of the study, there was no primary and secondary outcomes to analyze.
